# Karst dolines provide diverse microhabitats for different functional groups in multiple phyla

**DOI:** 10.1038/s41598-019-43603-x

**Published:** 2019-05-09

**Authors:** Zoltán Bátori, András Vojtkó, István Elek Maák, Gábor Lőrinczi, Tünde Farkas, Noémi Kántor, Eszter Tanács, Péter János Kiss, Orsolya Juhász, Gábor Módra, Csaba Tölgyesi, László Erdős, Dianne Joy Aguilon, Gunnar Keppel

**Affiliations:** 1 Department of Ecology, University of Szeged, Közép fasor 52, H-6726 Szeged, Hungary; 2 Department of Botany, Eszterházy Károly University of Applied Sciences, Eszterházy tér 1, H-3300 Eger, Hungary; 3 Museum and Institute of Zoology, Polish Academy of Sciences, Wilcza street 64, 00-679 Warsaw, Poland; 4 Aggtelek National Park Directorate, Tengerszem oldal 1, H-3758 Jósvafő, Hungary; 5 Department of Terrestrial Ecology, MTA Centre for Ecological Research, Alkotmány út 2-4, H-2163 Vácrátót, Hungary; 6 Doctoral School of Environmental Sciences, University of Szeged, Rerrich Béla tér 1, H-6720 Szeged, Hungary; 7 Doctoral School in Biology, Faculty of Science and Informatics, University of Szeged, Közép fasor 52, H-6726 Szeged, Hungary; 8 MTA-DE Functional and Restoration Ecology Research Group, Egyetem tér 1, H-4032 Debrecen, Hungary; 9 Department of Forest Biological Sciences, College of Forestry and Natural Resources, University of the Philippines Los Baños, 4031 Laguna, Philippines; 10 Natural and Built Environments Research Centre, School of Natural and Built Environments, University of South Australia, Mawson Lakes Campus, GPO Box 2471, Adelaide, South Australia 5001 Australia; 11 Future Industries Institute, University of South Australia, Mawson Lakes Campus, GPO Box 2471, Adelaide, South Australia 5001 Australia; 12 Biodiversity, Macroecology and Biogeography Group, Faculty of Forest Sciences and Forest Ecology, University of Goettingen, Göttingen, Germany

**Keywords:** Climate-change ecology, Biogeography

## Abstract

Fine-scale topographic complexity creates important microclimates that can facilitate species to grow outside their main distributional range and increase biodiversity locally. Enclosed depressions in karst landscapes (‘dolines’) are topographically complex environments which produce microclimates that are drier and warmer (equator-facing slopes) and cooler and moister (pole-facing slopes and depression bottoms) than the surrounding climate. We show that the distribution patterns of functional groups for organisms in two different phyla, Arthropoda (ants) and Tracheophyta (vascular plants), mirror this variation of microclimate. We found that north-facing slopes and bottoms of solution dolines in northern Hungary provided key habitats for ant and plant species associated with cooler and/or moister conditions. Contrarily, south-facing slopes of dolines provided key habitats for species associated with warmer and/or drier conditions. Species occurring on the surrounding plateau were associated with intermediate conditions. We conclude that karst dolines provide a diversity of microclimatic habitats that may facilitate the persistence of taxa with diverse environmental preferences, indicating these dolines to be potential safe havens for multiple phyla under local and global climate oscillations.

## Introduction

Environmental heterogeneity is often positively related to biodiversity^1–4^. Topography and biological structures can create microhabitats with unique microclimates^5–7^, which species may depend on for survival^8,9^. These microhabitats may be warmer, drier, cooler and/or moister than the prevailing regional climate^5,10^, creating a mosaic of microclimates that can allow species to survive changes in their environment by migrating short distances between microhabitats^10,11^. These fine-scale mosaics improve a species’ chances to persist in a landscape, calling into question the results of large-scale (resolutions ≥1 km^2^) species distribution models^7,9,12,13^. As a result, attempts to regionally model climatic data at 50–100 m resolution have been made^7,13^.

Furthermore, topographic complexity can create habitats that remain environmentally more stable through time, even as regional climate changes. Such habitats may therefore facilitate the persistence of biodiversity and are known as refugia^14–16^. Refugia are important for conservation planning and may offer the only chance of *in situ* survival for many species^17–19^. Microrefugia are small areas that provide such suitable pockets of relatively stable microclimate^20–22^. For instance, areas of high topographic convergence (e.g. local depressions and valleys)^23–25^ retain cooler microclimates when regional climates warm through drainage of cold air currents at night^26–28^.

Karst areas cover about 20% of the earth’s dry land surface^29^ and their complex topography provides various ecological niches for plants and wildlife, playing a crucial role in the maintenance of world’s biodiversity^30,31^. Karst systems support unique microclimates in several microhabitats, such as south- and north-facing slopes, enclosed depressions (‘dolines’) and ravines^21,32–34^. Where dolines are associated with cave entrances and/or sinks, the cave and/or sink will further influence the microclimate^35^. Several studies indicate that karst areas in Europe have supported the populations of both cool- and warm-adapted taxa outside their main distribution area (i.e. climate relicts) during past Quaternary climate oscillations^36–39^.

A number of studies indicate that dolines may play a crucial role in maintaining biodiversity. They harbour unique taxa that are rare or absent in the surrounding areas (e.g. endemic and relict species)^40–42^ and are characterised by high plant, genetic and habitat diversity^43–46^. Cool-adapted species from various phyla (e.g. Arthropoda, Bryophyta, Mollusca and Tracheophyta) have been documented from dolines^47–50^. In addition, dolines may also provide key habitats for warm-adapted species^35^. Documenting the distribution of both cool- and warm-adapted taxa with respect to microclimate inside and adjacent to dolines may therefore help us to better understand the potential of dolines to function as microrefugia.

Here we investigate the distribution patterns of organisms in four different microhabitats inside (south-facing slopes, bottoms and north-facing slopes) and outside of dolines (plateau) in the Bükk Mountains, northern Hungary. We illustrate that dolines can support a wide range of microclimatic conditions (both warmer and cooler than the surrounding plateau) and that they have the capacity to support diverse ant and plant assemblages. Furthermore, we show that the distributions of different functional groups (cool- and moist-adapted *versus* warm- and dry-adapted) of both, ants and plants, respond to the fine-scale microclimatic differences among the microhabitats in a similar manner. Therefore, our results show that dolines can be crucial safe havens for species from various phyla and highlight that investigating climate change responses will require high (~10 m) resolution environmental data for some taxa.

## Results

### Microclimate

**Figure 1:**
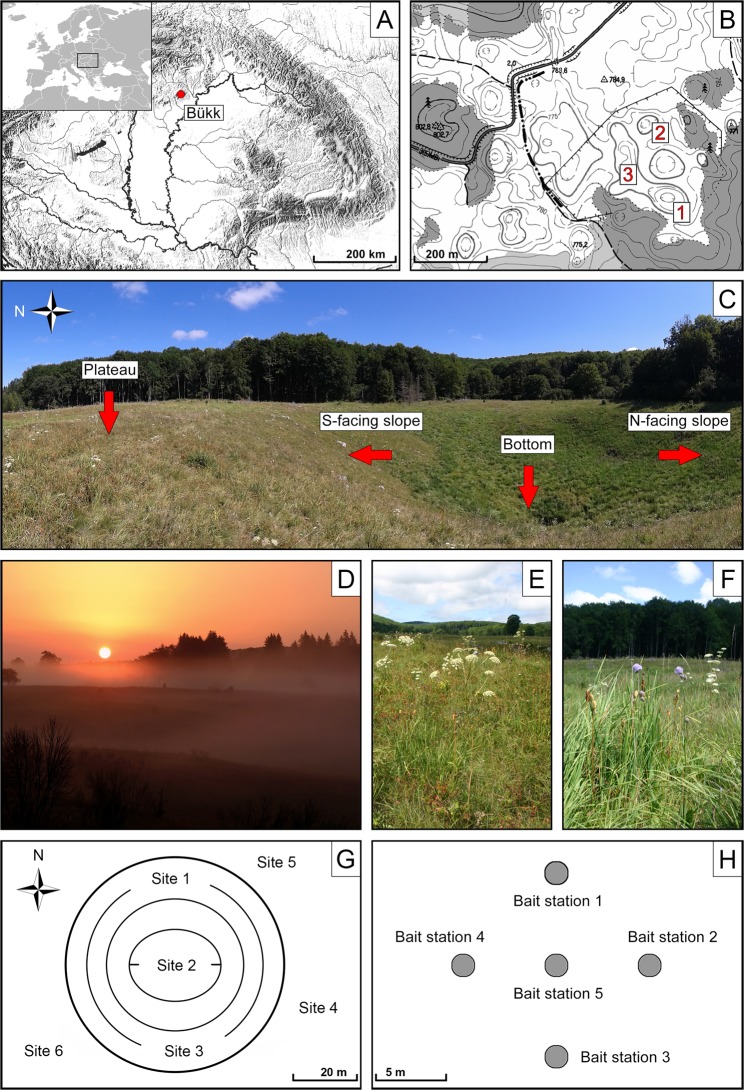
Study sites and study design. (**A**) Location of the Bükk Mountains in Hungary. (**B**) Study area and studied karst dolines (1–3). (**C**) Different parts of doline 1. (**D**) Early morning fog in dolines. (**E**) Semi-dry grassland on the plateau between dolines. (**F**) Wet meadow in the bottom of a doline. (**G**) Location of the study sites (site 1–6) in and around a doline. (**H**) Set-up of bait stations in a cross-shaped pattern.

**Figure 2:**
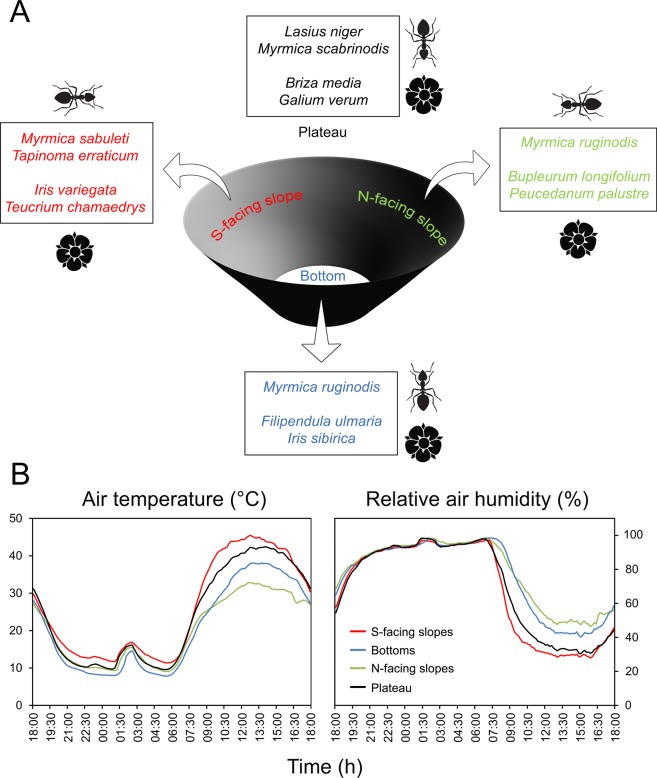
Schematic illustration of the differentiation of microhabitats within karst dolines in Bükk, Hungary, with regard to (**A**) abundant ant and plant taxa and (**B**) microclimate. North-facing slopes and bottoms of dolines are consistently cooler and moister, while south-facing slopes are consistently warmer and drier than their surroundings. Abundant taxa of both phyla differ among microhabitats.

Temperatures were higher on south-facing slopes (T_24_ = 25.9 °C; T_d_ = 33.8 °C; T_n_ = 13.8 °C) than in other microhabitats (Figs 1 and 2B). The mean daily temperature was similar on north-facing slopes (T_24_ = 20.1 °C) and in doline bottoms (T_24_ = 20.6 °C), while night temperatures were lowest in bottoms (T_n_ = 9.78 °C). Night temperatures were similar on north-facing slopes (T_n_ = 11.5 °C) and the plateau (T_n_ = 11.8 °C). The mean daytime temperature was higher in bottoms (T_d_ = 27.7 °C) than on north-facing slopes (T_d_ = 25.7 °C). Temperatures on the plateau were intermediate (T_24_ = 24.0 °C; T_d_ = 31.8 °C; T_n_ = 11.8 °C). Mean daily relative humidity was lowest on south-facing (RH_24_ = 68.0%) and highest on north-facing slopes (RH_24_ = 78.4%). The values were intermediate in bottoms (RH_24_ = 76.2%) and on the plateau (RH_24_ = 70.0%). At night, relative humidity was similar (RH_n_ = 92.9–94.0%) in all microhabitats. However, daytime relative humidity was usually higher on north-facing slopes (RH_d_ = 68.2%) than in bottoms (RH_d_ = 65.2%), on south-facing slopes (RH_d_ = 51.7%) and on the plateau (RH_d_ = 55.1%). A small, intermittent temperature increase in all microhabitats was recorded around 1:30 hours and indicated an inflow of warmer air from the surrounding lower altitudes.

### Species composition

A total of 14 ant (nine from baiting and another five from hand collecting) and 145 plant species (from the plots) were recorded in our sites (Fig. 1G; Supplementary Tables S1 and S2). Hand collection yielded 52 ant individuals, 13 were found on south-facing slopes, five in bottoms, 11 on north-facing slopes and 23 on the plateau. In terms of diagnostic species, south-facing slopes had two ant and 13 plant species, while two ant and 20 plant species were identified for the plateau. North-facing slopes and bottoms had 15 and nine plant species, respectively, and one ant species (Fig. 2A; Table 1). Dry grasslands dominated the south-facing slope of dolines 1 and 3, while semi-dry grasslands covered the south-facing slope of doline 2 and the major part of the plateau. North-facing slopes and doline bottoms were dominated by wet meadows.

**Table 1:** Synoptic table of ants and plants associated with different microhabitats (south-facing slopes, bottoms and north-facing slopes of dolines, and the plateau) in Bükk (Hungary).

Ants	Plants
**S-facing slopes**	
*Tapinoma erraticum* (0.30), *Myrmica sabuleti* (0.79)	*Polygonatum odoratum* (0.32), *Digitalis grandiflora* (0.39), *Waldsteinia geoides* (0.40), *Brachypodium pinnatum* (0.43), *Anemone sylvestris* (0.47), *Sedum maximum* (0.50), *Verbascum austriacum* (0.51), *Festuca rupicola* (0.52)*, *Origanum vulgare* (0.53), *Fragaria viridis* (0.55)*, *Geranium sanguineum* (0.61), *Teucrium chamaedrys* (0.66), *Iris variegata* (0.83)
**Bottoms**	
*Myrmica ruginodis* (0.25)*	*Potentilla erecta* (0.35), *Filipendula ulmaria* (0.38), *Iris sibirica* (0.39), *Festuca ovina* (0.40), *Agrostis canina* (0.45), *Aconitum variegatum* s.l. (0.46)*, *Molinia caerulea* (0.50), *Geranium palustre* (0.51), *Urtica dioica* (0.57)
**N-facing slopes**	
*Myrmica ruginodis* (0.41)*	*Thalictrum lucidum* (0.32), *Senecio integrifolius* (0.32), *Stellaria holostea* (0.32), *Galium mollugo* (0.36), *Primula elatior* (0.36), *Aconitum variegatum* s.l. (0.38)*, *Aconitum moldavicum* (0.40), *Bupleurum longifolium* (0.40), *Carex pilosa* (0.40), *Peucedanum palustre* (0.45), *Astrantia major* (0.46), *Euphorbia lucida* (0.48), *Calamagrostis arundinacea* (0.52), *Aegopodium podagraria* (0.52), *Luzula luzuloides* (0.57)
**Plateau**	
*Lasius niger* (0.29), *Myrmica scabrinodis* (0.34)	*Ranunculus polyanthemos* (0.32), *Valeriana officinalis* subsp. *collina* (0.32), *Cirsium pannonicum* (0.34), *Carex michelii* (0.35), *Koeleria pyramidata* (0.35), *Linum catharticum* (0.35), *Primula veris* (0.37), *Galium verum* (0.37), *Seseli libanotis* (0.39), *Poa pratensis* s.l. (0.41), *Centaurea scabiosa* subsp. *sadleriana* (0.42), *Filipendula vulgaris* (0.42), *Helictotrichon alpinum* (0.44), *Phleum phleoides* (0.46), *Briza media* (0.47), *Carex filiformis* (0.47), *Thesium linophyllon* (0.49), *Festuca rupicola* (0.52)*, *Fragaria viridis* (0.55)*, *Hypericum perforatum* (0.58)

Within blocks, species are listed by increasing values of the phi (*Φ*) coefficient of association between species and habitat (in parenthesis). Four of the species, marked with an asterisk, were diagnostic for two different microhabitats.

**Figure 3:**
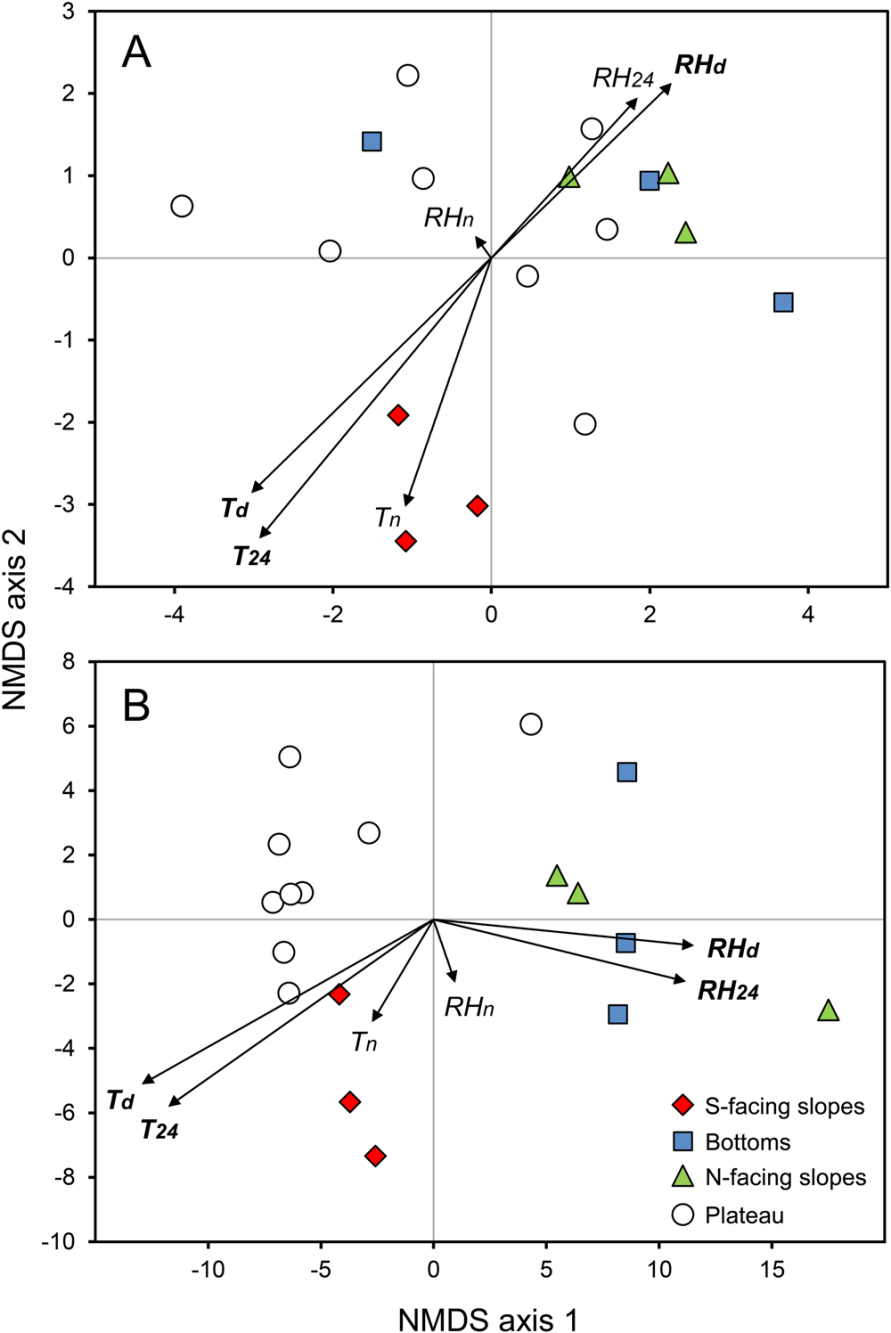
Non-metric multidimensional scaling (NMDS) ordination for (**A**) ant and (**B**) plant assemblages in different microhabitats (south-facing slopes, bottoms and north-facing slopes of dolines, and the plateau) with fitted vectors of mean daily temperature (T_24_) and relative humidity (RH_24_), mean daytime temperature (T_d_) and relative humidity (RH_d_), and mean night temperature (T_n_) and relative humidity (RH_n_). Vector length indicates the strength of correlation (see Supplementary Table S3). Microclimate variables that were significantly correlated to the ordination (T_24_, T_d_, RH_24_ and RH_d_) are indicated in boldface.

NMDS ordinations of bait stations (stress factor: 0.119) and vegetation plots (stress factor: 0.064) showed that the species composition of ant and plant assemblages differed among microhabitats (Fig. 3A,B). These differences were significant, except between ant assemblages of bottoms and north-facing slopes (PERMANOVA; Table 2). Plant assemblages of all microhabitats displayed highly significant differences (*p* < 0.001). T_24_, T_d_ and RH_d_ were significantly related to the ordination of ant, and T_24_, T_d_, RH_24_ and RH_d_ to the ordination of plant assemblages (Supplementary Table S3), with assemblages on south-facing slopes being associated with higher temperatures and lower humidity, and assemblages on north-facing slopes and in doline bottoms being related to lower temperatures and higher humidity.

**Table 2:** Comparisons of the ant and plant assemblages in different microhabitats (south-facing slopes, bottoms and north-facing slopes of dolines, and the plateau) with permutational multivariate analysis of variance (PERMANOVA).

	Ants			Plants		
	*F*	*R* ^2^	*p*	*F*	*R* ^*2*^	*p*
S-facing slopes vs. Bottoms	20.96	0.327	**<0**.**001**	10.88	0.279	**<0**.**001**
S-facing slopes vs. N-facing slopes	15.70	0.389	**<0**.**001**	7.82	0.349	**<0**.**001**
S-facing slopes vs. Plateau	11.37	0.171	**<0**.**001**	5.34	0.169	**<0**.**001**
N-facing slopes vs. Bottoms	1.35	0.034	0.261	2.86	0.164	**<0**.**001**
N-facing slopes vs. Plateau	8.83	0.141	**<0**.**001**	6.84	0.204	**<0**.**001**
Bottoms vs. Plateau	4.89	0.071	**0**.**004**	5.08	0.237	**<0**.**001**

The *p* values were corrected with the FDR (false discovery rate) method. Significant differences are indicated by bold *p* values.

### Functional groups of species

**Figure 4:**
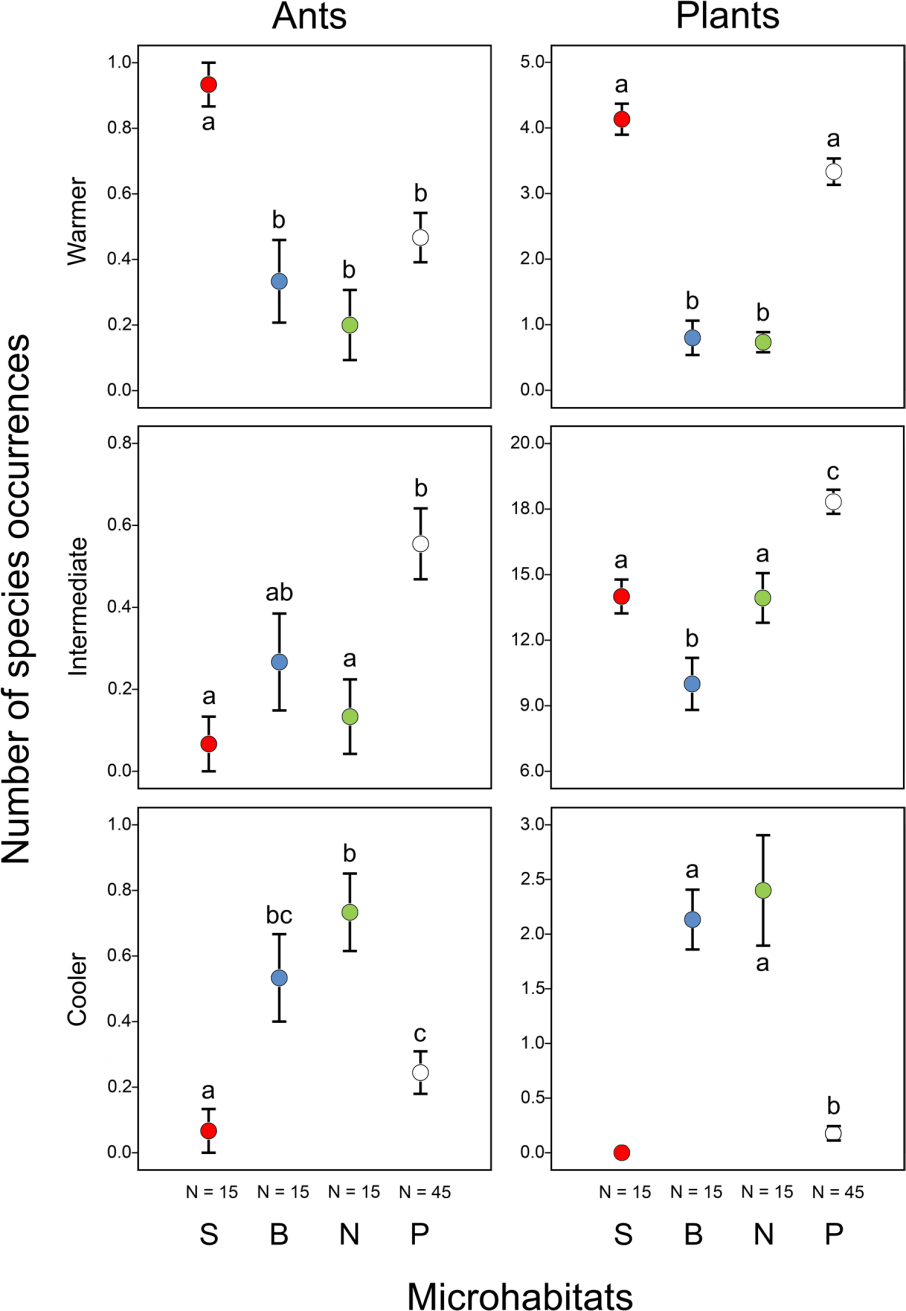
Occurrences of ant and plant species (mean ± SE) belonging to different functional groups of temperature requirements (warmer, intermediate and cooler) in different microhabitats (S: south-facing slopes, B: bottoms and N: north-facing slopes of dolines, and P: the plateau). Significant differences detected using mixed-effect models (see Supplementary Table S4) are indicated by different lower case letters (a–c).

**Figure 5:**
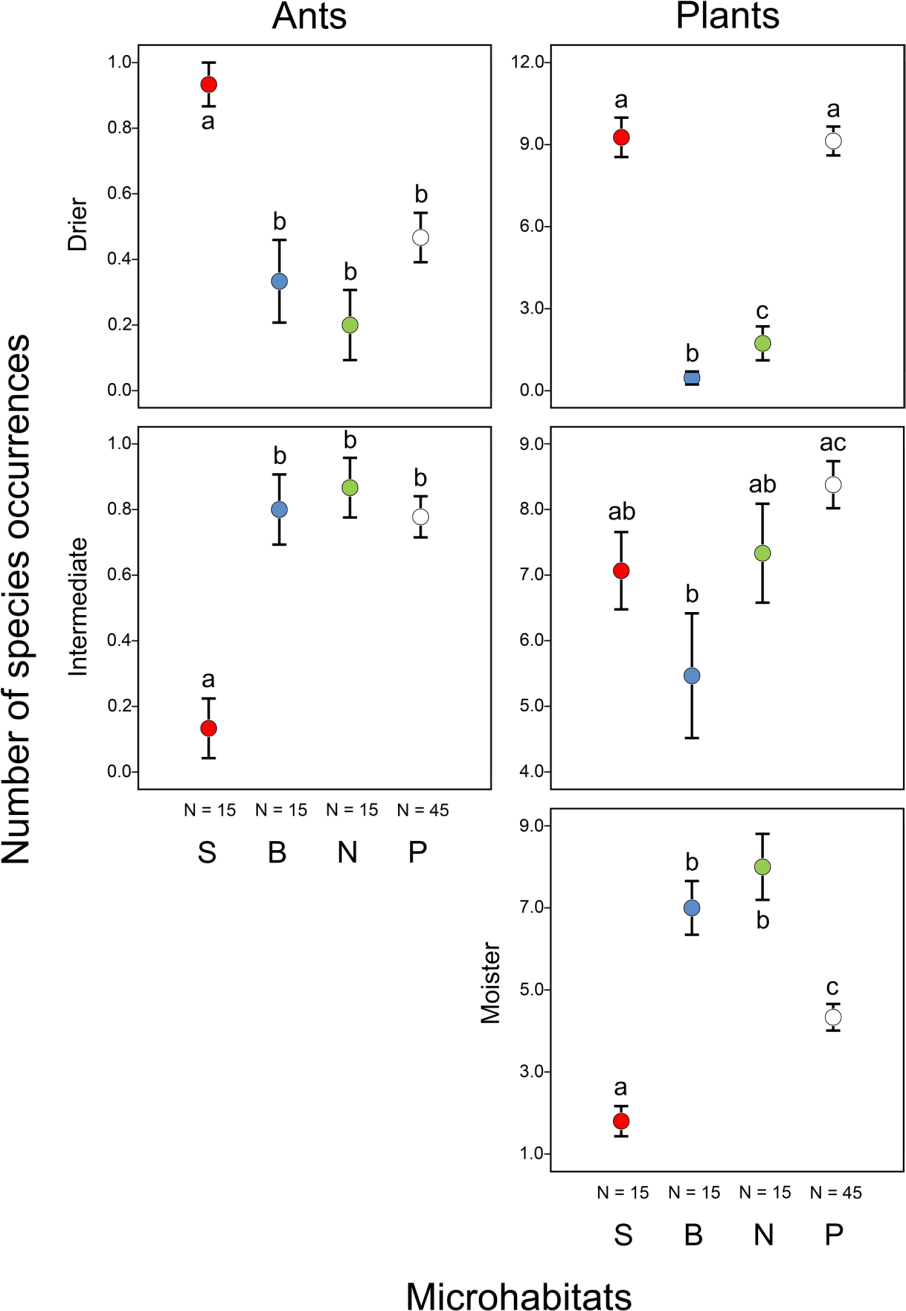
Occurrences of ant and plant species (mean ± SE) belonging to different functional groups of moisture requirements (drier, intermediate and moister) in different microhabitats (S: south-facing slopes, B: bottoms and N: north-facing slopes of dolines, and P: the plateau). Significant differences detected using mixed-effect models (see Supplementary Table S5) are indicated by different lower case letters (a–c).

Functional groups of both ants and plants showed significant preferences for certain microhabitats (Figs 4 and 5; Supplementary Tables S4 and S5). On north-facing slopes, 64% of the hand-collected ants (individuals) were species adapted to cooler conditions, and 82% of the individuals were species adapted to intermediate moisture conditions. In other microhabitats, most of the hand-collected ants (100% on south-facing slopes, 80% in bottoms and 52% on the plateau) were species adapted to warmer and drier conditions. Ant (collected from bait stations) and plant species adapted to warmer and/or drier conditions occurred more frequently on south-facing slopes than on north-facing slopes and in doline bottoms (Figs 4 and 5; Supplementary Tables S4 and S5). Conversely, ant and plant species adapted to cooler and/or moister conditions were generally most frequent on north-facing slopes and in doline bottoms compared to south-facing slopes and the plateau. We did not find any ant species adapted to moister conditions and no plant species adapted to cooler conditions on south-facing slopes.

## Discussion

Karst dolines are topographically complex environments that provide a variety of microhabitats. We have demonstrated that these habitats can be cooler and moister or warmer and drier than the surrounding plateau, providing high environmental heterogeneity at very fine scales. We further documented that the fine-scale distributions of functional groups in two different phyla (Arthropoda and Tracheophyta) correspond to these different microhabitats. To our knowledge, this is the first study to illustrate that the fine-scale topography of dolines provides microhabitats for diverse functional groups (cool- and moist-adapted *versus* warm- and dry-adapted) of both ants and vascular plants within tens of meters.

Topographic complexity increases the climatic variability in an area over fine scales^24,51,52^. Despite recording temperature and relative humidity for only a relatively short period of time (24 hours), we found that karst dolines introduce great variation in microclimates. For instance, mean daytime temperatures were more than 8 °C warmer on south- than on north-facing slopes (Fig. 2B). Previous microclimatic studies over longer time periods, i.e. from a few days to a year, also indicate north-facing slopes and bottoms of dolines to be cooler and more humid than south-facing slopes and the surrounding plateau^23,35,53–56^. In addition to receiving less solar radiation, doline bottoms tend to receive more water^57^, likely contributing to the higher relative humidity and lower temperatures recorded in these habitats^58,59^. North-facing slopes and bottoms of dolines may retain snow cover longer than south-facing slopes and plateaus^60^.

Fine-scale variation in environmental conditions affects the distributions of organisms^61–63^. Both ant and vascular plant species responded to microclimatic variation in our study. The cooler and moister north-facing slopes and bottoms of dolines in Bükk acted as key habitats for plants adapted to cooler and/or moister conditions (e.g. *Bupleurum longifolium* and *Iris sibirica*) and ants adapted to cooler conditions (e.g. *Myrmica ruginodis*) (Fig. 2A). On the other hand, south-facing slopes acted as key habitats both for ants (e.g. *Myrmica sabuleti*) and plants (e.g. *Iris variegata*) adapted to warmer and/or drier conditions, while many ant (e.g. *Lasius niger*) and plant species (e.g. *Galium verum*) found on the plateau indicated intermediate temperature and/or moisture conditions (Fig. 2A). The ant species recorded are not known to form strict trophic relationships with plants, and the main drivers of the observed patterns seem to be their temperature and moisture preferences. Our results underline the importance of considering fine-scale environmental variation when investigating the distribution of biodiversity^63,64^. Even the highest resolution (50–100 m) climate data currently available^7,13^ would likely be insufficient to detect the environmental heterogeneity provided by the karst dolines in our study, which are <200 m wide and <20 m deep.

We have documented a strong, concerted response to fine-scale topography by the distributions of microclimate and species in two major taxonomic groups (ants, Arthropoda; and vascular plants, Tracheophyta). However, other taxa also display distribution patterns reflecting changes in environmental conditions in dolines over short distances. For instance, the increased soil moisture content on north-facing slopes and bottoms of solution dolines in the Aggtelek Karst area (Hungary) has been shown to provide suitable habitats for several cool-adapted species of land snails (Mollusca)^49^ and terrestrial isopods (Arthropoda)^65^. Dolines in Mexico, Australia and China (‘cenotes’ and ‘tiankengs’) have also been shown to maintain populations of rare taxa in various phyla^46,66–68^, highlighting dolines as important safe havens for biodiversity on a global scale.

Species may respond to climate changes by range-shifting^69^ or by persisting in environmentally stable habitats^70,71^. In karst dolines, species could potentially track suitable microclimates over a wide range of regional climatic changes with minimal movement because cooler/moister and warmer/drier microclimates vary over very short distances. As few areas can buffer opposing trends in environmental conditions^72^, karst dolines may be particularly important for maintaining biodiversity through time. Therefore, they could be considered high-capacity microrefugia^73^. The highest-capacity microrefugia for cool-adapted taxa can usually be found in cold, humid and topographically complex areas^74,75^.

Therefore, karst dolines may play important roles in facilitating the persistence of different phyla under global warming, which poses a serious threat to global biodiversity^76^. Regional predictions of climate change suggest that warming in East-Central Europe will continue in the coming decades^77^. These changes are already impacting the distributions of ants^78^ and vascular plants^79^ in a sand-dune area in Hungary, with drought-tolerant species replacing dune slack species over the last decades. Therefore, species adapted to warmer and/or drier conditions are expected to expand their distribution from south-facing slopes of dolines to surrounding areas. However, north-facing slopes and bottoms of dolines could provide important microrefugia from global warming by facilitating the persistence of species adapted to cooler and/or moister and to intermediate conditions. The retention of cooler microclimates in these habitats may be facilitated by lower solar radiation, thicker soil layer, higher soil moisture and cool-air pooling^27,58,80^.

Although our data supports dolines to be safe havens for relict plant species (e.g. *Aconitum variegatum*, *Bupleurum longifolium* and *Dracocephalum ruyschiana* in Bükk) in the current climate, future studies should aim to confirm the status of dolines as refugia (i.e. places providing environmental conditions that are comparatively stable over long time periods) under global warming. While our 24-hour data demonstrate that dolines are currently providing cooler and warmer microclimates than the surrounding plateau, this does not necessarily prove stability and long-term monitoring would be needed for this. Available data from north-facing slopes in the Northern Hemisphere does suggest that such habitats undergo slower changes under global warming^81,82^. Alternatively, the microclimate of dolines could be investigated along a temperature gradient using a space-for-time substitution approach to determine if north-facing slopes and bottoms of dolines indeed retain more stable microclimates. Finally, functional traits can be reflective of long-term environmental stability and therefore could provide important eco-evolutionary information about refugia^83^.

We conclude that enclosed depressions in karst surfaces provide a diversity of microclimates that have the potential to enable the persistence of various taxa in different phyla and under various climatic trends. These dolines may be vital for facilitating the *in situ* persistence of numerous species under local and global climate oscillations. This implies that modelling of climate change impacts on the distribution of biodiversity will need to consider fine-scale topographic variation occurring within tens of meters to arrive at accurate predictions.

## Methods

### Study area

Our study area was located on the karst plateau of the Bükk Mountains (48°04′31″N, 20°29′57″E), in northern Hungary, at an altitude of approximately 780 m (Fig. 1A,B) in the beech (*Fagus sylvatica*) forest zone. This mountain range is believed to be an important refugial area in Hungary, supporting relict plant populations from both warmer (e.g. *Clinopodium thymifolium*, *Cotinus coggygria* and *Ferula sadleriana*) and cooler (e.g. *Aconitum variegatum*, *Bupleurum longifolium* and *Dracocephalum ruyschiana*) periods^84,85^. The plateau has a cool and humid climate, with a mean annual temperature of 6.3 °C and a mean annual precipitation of 800 mm. The plateau has typical karst landform features, such as solution dolines^86–88^, with a bowl-shaped geometry (Fig. 1C) and unique microclimate. At night, cold-air pooling occurs in these depressions, and the occurrence of frost or fog is possible all year round^55^ (Fig. 1D). According to some researchers^89^, the coldest areas in Hungary can be found in the non-forested dolines of Bükk. Previous investigations showed that north-facing slopes of these dolines are consistently cooler and moister than the surrounding plateau, and that south-facing slopes provide the warmest microhabitats^54,90^. The study area is well known for its unique wildlife and is part of the strictly protected area network of the Bükk National Park. The entire area is fenced to prevent overgrazing, soil erosion and the illegal collection of wild plants. Semi-dry grasslands and wet meadows are the dominant vegetation types within the fenced area^91^ (Fig. 1E,F).

### Sampling design

Three large solution dolines were selected (Fig. 1B,C). Dolines 1 and 2 had diameters of 100 and 70 m and depths of 17 and 15 m, respectively. The longer diameter of doline 3 was 190 m, while the shorter was 65 m, its depth was 13 m. Six sampling sites were established per each doline (18 sites in total), one on the south-facing slope, one in the bottom, one on the north-facing slope and three on the surrounding plateau. The sites were located at least 20 m from each other (Fig. 1G). Sampling and microclimate measurements were carried out in August, under clear weather conditions, at the peak of the growing season.

Ants and plants were selected as focal taxa because ant colonies and plants share many similarities^92^. Both groups usually ‘nest’ in or on the ground and use their modules (e.g. plant roots and ant workers) to forage in the surrounding habitat^93,94^. In addition, due to the relatively fixed location of ant colonies and plants, competition in both groups is confined to well-defined zones. Similarities also exist in their functional roles in a given community (e.g. subordinate, specialist and cryptic species). Finally, ant foundresses (i.e. colony-founding queens) can be considered analogous to dispersing plant seeds^93,95^.

Considering that the study area is a strictly protected nature reserve, we used only non-destructive sampling methods such as baiting and hand collecting to assess the species diversity and relative abundance of ants. At each site, we placed five bait stations in a cross-shaped pattern at 5-m intervals (90 bait stations in total) (Fig. 1H). Baits were plastic discs (8 cm in diameter) with a quarter-teaspoon of a mixture of tuna and honey as a food reward. Foraging activity on baits was monitored every 40 minutes from 7:00 to 9:40, overlapping with the daily period of peak ant activity. During each observation, we recorded the presence and number of workers of each ant species on the bait. Baits were replenished as necessary. In addition, we also performed hand collecting to sample those ant species that may have not visited the baits. We visually searched the ground surface in each site for 5 minutes, hand collecting any individuals (workers, incipient queens, etc.) found. Ants were identified to morphospecies or genus level in the field, and representatives were collected and preserved in 95% ethanol for later species determination^96^. Field-collected specimens were identified in the laboratory using the keys of Seifert^97^ and Czechowski *et al*.^98^. All the collected specimens were deposited at the Department of Ecology, University of Szeged. We used Bolton’s catalogue^99^ and the Hymenoptera Name Server^100^ to determine the valid names of all ant species.

For plants, five randomly selected 1 m × 1 m plots were established in each site (90 plots in total). We recorded the presence/absence of all vascular plant species in all plots. Nomenclature follows ‘The Plant List’ (www.theplantlist.org).

To provide information on the microclimate of the study area, air temperature (T) and relative air humidity (RH) were recorded every minute for 24 hours using Voltcraft DL-121TH data loggers. Sensors were suspended 10 cm above the ground to allow sufficient wind flow to ensure that no humidity was trapped by the sensor casing and actual air temperature and humidity were measured.

### Species grouping

We classified all ant and plant species according to their temperature and moisture requirements following the methods of Czechowski *et al*.^98^ for ants and Borhidi^101^ for plants (Supplementary Table S6 and Table S7). These were reduced to six functional groups applied to both ants and plants: (1) species adapted to warmer conditions, (2) species adapted to cooler conditions, (3) species adapted to intermediate temperature conditions, (4) species adapted to drier conditions, (5) species adapted to moister conditions, and (6) species adapted to intermediate moisture conditions (see details in Supplementary Tables S6 and S7). We did not analyse combined groups (temperature + moisture) because that would have made the interpretation of results difficult (with many groups), especially in the case of plants. All six main groups of plants and five main groups of ants (groups 1–4 and 6; ants adapted to moister conditions were absent in our study) were analysed. Because the temperature and moisture requirements of ant and plant species are not the same, direct comparisons between them were not possible.

### Data analysis

The temperature and relative humidity data were averaged over 10-minute intervals across all sites of south-facing slopes, bottoms, north-facing slopes and the plateau, respectively, and plotted using a line graph. Extreme environmental values are generally more informative regarding the distribution of organisms, but maximum relative humidity values in our case often reached 100%, therefore we considered mean values more suitable for differentiating between the microclimatic properties of microhabitats than extreme values.

From the site-averaged data, we calculated the mean daily temperature (T_24_) and relative humidity (RH_24_), mean daytime temperature (T_d_) and relative humidity (RH_d_), and mean night temperature (T_n_) and relative humidity (RH_n_). We also calculated these microclimate variables separately for each site, and used them in multivariate analyses.

The diagnostic ant and plant species of the microhabitats were determined by calculating the phi (*Φ*) coefficient of association between species and habitat. Calculations for ants were based on data from bait stations. Species with *Φ* > 0.2 were considered diagnostic for ants and species with *Φ* > 0.3 for plants. Different threshold values for ants and plants were used due to the differences in the total number of species within each of these taxonomic groups. Non-diagnostic species were excluded with Fisher’s exact test (*p* < 0.05) following Tichý and Chytrý^102^. Fidelity measures were calculated using the JUICE program^103^.

We used permutational multivariate analysis of variance (PERMANOVA) to test the effect of microhabitats (south-facing slopes, bottoms, north-facing slopes and the plateau) on the species composition of ant and plant assemblages. We used the raw presence/absence data of species for each sampling plot in the source matrices. We applied the Jaccard dissimilarity, performed 5000 permutations and also accounted for the nested design of the data set. When a PERMANOVA yielded significant results, we calculated pairwise PERMANOVAs among the microhabitat types. PERMANOVAs were calculated in R^104^ using the *adonis* function of the ‘vegan’ package^105^. We used the FDR (false discovery rate) method to adjust *p* values for multiple comparisons (*p*.*adjust* function). We prepared non-metric multidimensional scaling (NMDS) ordinations to visually illustrate compositional differences. To remove the confounding effect of the nested data structure on the resulting point clouds, we lumped data from the different sampling plots of each site of each doline together and used the frequency data, ranging from one to five, of the species in the source matrices. We used Euclidean distances and two dimensions (after assessing stress factors for one to five dimensions). NMDS ordinations were done using the metaMDS function of the ‘vegan’ package. To assess the relationships between microclimate variables (T_24_, RH_24_, T_d_, RH_d_, T_n_ and RH_n_) and species assemblages, we fitted environmental vectors onto the ordination space using the *envfit* function and calculated correlations between ordination values and fitted vectors.

We used mean-and-whisker plots to illustrate the distribution of the various functional groups in different microhabitats. To test if these differences were significant we used generalized linear mixed-effects models (GLMM). Calculations for ants were based on data from bait stations. Five models were built for ants (three for temperature and two for moisture) and six models for plants (three for temperature and three for moisture). In the full models, different microhabitats were included as fixed factors, the number of ant and plant species as dependent variables, and location (i.e. doline 1, 2 and 3) as the random factor. We transformed the data of ants to binary scale (presence/absence) and used a binomial error term because each functional group had a high preference for one or a few sites and were very rare in other sites, leading to zero inflation of the data. No transformation was needed for plants, and we used Poisson or, if overdispersion was detected, negative binomial error term. GLMMs were performed in R using the *glmer* function of the ‘lme4’ package^106^. Full models were tested for significance with analysis of variance, using the *Anova* function of the ‘car’ package^107^. Pairwise comparisons of factor levels were undertaken with the *relevel* function and the FDR method (*p*.*adjust* function) was used to correct *p* values for multiple comparisons.

## Supplementary information

Supplementary Dataset 1

## Data Availability

The datasets generated and/or analysed during the current study are available from the corresponding author on reasonable request.
