# Supplementary material for: Karst dolines provide diverse microhabitats for different functional groups in multiple phyla
Source: Sci Rep. 2019 May 9;9:7176. doi: 10.1038/s41598-019-43603-x (PMC6509348; doi:10.1038/s41598-019-43603-x)
Supplement: Supplementary file 1 — Supplementary Dataset 1 [file 41598_2019_43603_MOESM1_ESM.pdf]

Karst dolines provide diverse microhabitats for different functional groups in multiple phyla

Zoltán Bátori*, András Vojtkó, István Elek Maák, Gábor Lőrinczi, Tünde Farkas, Noémi Kántor, Eszter Tanács, Péter János Kiss, Orsolya Juhász, Gábor Módra, Csaba Tölgyesi, László Erdős, Dianne Joy Aguilon, Gunnar Keppel

*zbatory@gmail.com

Table S1 List of ant species of our study area with the temperature and moisture requirements of species (see Table S6 for definition of categories)

	**Temperature**	**Moisture**
Formica sanguinea	warmer	drier
Formica truncorum	warmer	drier
*Formicoxenus nitidulus**	-	-
Lasius niger	intermediate	intermediate
Lasius platythorax	cooler	intermediate
*Leptothorax acervorum***	-	-
Myrmica lobicornis	cooler	intermediate
Myrmica ruginodis	cooler	intermediate
Myrmica sabuleti	warmer	drier
Myrmica scabrinodis	intermediate	intermediate
Tapinoma erraticum	warmer	drier
Temnothorax albipennis	warmer	drier
Temnothorax nigriceps	warmer	drier
Tetramorium cf. *caespitum*	warmer	drier

*: inquiline ant that parasitizes *Formica* species
**: euryoecious species (able to live under variable conditions)

Table S2 List of pant species of our study area with the temperature and moisture requirements of species (see Table S7 for the definition of categories)

	Temperature	Moisture
Achillea collina	intermediate	drier
Aconitum moldavicum	cooler	moister
Aconitum variegatum s.l.	cooler	moister
Aegopodium podagraria	intermediate	moister
Agrostis canina	intermediate	moister
Ajuga genevensis	intermediate	intermediate
Alchemilla monticola	cooler	moister
Allium oleraceum	warmer	drier
Anemone sylvestris	intermediate	drier
Anthoxanthum odoratum	intermediate	intermediate
Arabis hirsuta	intermediate	drier
Arrhenatherum elatius	intermediate	intermediate
Asperula cynanchica	warmer	drier
Astrantia major	cooler	moister
Brachypodium pinnatum	intermediate	intermediate
Briza media	intermediate	moister
Bromus erectus	intermediate	drier
Bupleurum longifolium	cooler	intermediate
Calamagrostis arundinacea	intermediate	intermediate
Calamagrostis epigejos	intermediate	intermediate
Campanula glomerata	intermediate	intermediate
Campanula persicifolia	intermediate	intermediate
Carduus acanthoides	intermediate	drier
Carex divulsa	intermediate	intermediate
Carex filiformis	intermediate	moister
Carex michelii	warmer	intermediate
Carex montana	intermediate	intermediate
Carex pallescens	intermediate	moister
Carex pilosa	intermediate	intermediate
Carlina acaulis	intermediate	intermediate
Centaurea jacea agg.	intermediate	intermediate
Centaurea phrygia subsp. *indurata*	warmer	intermediate
Centaurea scabiosa subsp. *sadleriana*	warmer	drier
Cirsium arvense	intermediate	intermediate
Cirsium pannonicum	warmer	intermediate
Clinopodium acinos	intermediate	drier
Colchicum autumnale	intermediate	moister
Cruciata glabra	intermediate	moister
Dactylis glomerata	intermediate	moister
Dianthus giganteiformis subsp. *pontederae*	warmer	drier
Digitalis grandiflora	intermediate	intermediate

Euphorbia cyparissias	intermediate	drier
Euphorbia lucida	intermediate	moister
Festuca ovina	cooler	intermediate
Festuca pratensis	intermediate	moister
Festuca rubra	intermediate	intermediate
Festuca rupicola	warmer	drier
Filipendula ulmaria	cooler	moister
Filipendula vulgaris	intermediate	intermediate
Fragaria viridis	intermediate	drier
Galium boreale	cooler	moister
Galium mollugo	intermediate	intermediate
Galium verum	intermediate	intermediate
Gentiana cruciata	intermediate	drier
Gentiana pneumonanthe	intermediate	moister
Geranium palustre	intermediate	moister
Geranium sanguineum	intermediate	drier
Helianthemum ovatum	intermediate	drier
Helictotrichon alpinum	intermediate	intermediate
Helictotrichon pubescens	intermediate	intermediate
Helleborus purpurascens	intermediate	intermediate
Hieracium sabaudum	intermediate	intermediate
Holcus lanatus	intermediate	moister
Hypericum perforatum	intermediate	drier
Hypochaeris maculata	intermediate	intermediate
Inula hirta	intermediate	drier
Iris graminea	warmer	intermediate
Iris sibirica	intermediate	moister
Iris variegata	warmer	intermediate
Jacobaea vulgaris	intermediate	drier
Koeleria pyramidata	intermediate	drier
Lathyrus pratensis	intermediate	moister
Leucanthemum vulgare	intermediate	moister
Linaria vulgaris	intermediate	drier
Linum catharticum	intermediate	moister
Lotus corniculatus	intermediate	intermediate
Luzula campestris	intermediate	intermediate
Luzula luzuloides	intermediate	intermediate
Medicago falcata	intermediate	drier
Molinia caerulea	intermediate	intermediate
Myosotis sylvatica	intermediate	moister
Origanum vulgare	intermediate	drier
Peucedanum palustre	intermediate	moister
Phleum phleoides	intermediate	drier
Pimpinella saxifraga	intermediate	drier
Plantago lanceolata	intermediate	intermediate

Plantago media	intermediate	intermediate
Poa pratensis s.l.	intermediate	moister
Polygonatum odoratum	warmer	drier
Polygonatum verticillatum	cooler	moister
Potentilla erecta	cooler	moister
Potentilla thuringiaca	intermediate	drier
Primula elatior	intermediate	moister
Primula veris	intermediate	intermediate
Prunella grandiflora	intermediate	intermediate
Prunella vulgaris	intermediate	moister
Prunus spinosa	intermediate	drier
Pulsatilla grandis	warmer	drier
Ranunculus acris	intermediate	moister
Ranunculus auricomus	intermediate	moister
Ranunculus polyanthemos	intermediate	intermediate
Rhamnus cathartica	intermediate	intermediate
Rosa gallica	warmer	drier
Rosa spinosissima	intermediate	intermediate
Rumex acetosa	intermediate	intermediate
Salvia pratensis	intermediate	drier
Sanguisorba minor	intermediate	drier
Sanguisorba officinalis	intermediate	moister
Scorzoneroides autumnalis	intermediate	moister
Securigera varia	intermediate	intermediate
Sedum maximum	intermediate	drier
Senecio integrifolius	intermediate	intermediate
Serratula tinctoria	intermediate	intermediate
Seseli libanotis	intermediate	drier
Silene vulgaris	intermediate	intermediate
Stachys officinalis	intermediate	intermediate
Stellaria holostea	intermediate	intermediate
Succisa pratensis	intermediate	moister
Symphytum tuberosum	intermediate	intermediate
Tanacetum corymbosum	intermediate	intermediate
Teucrium chamaedrys	intermediate	drier
Thalictrum lucidum	intermediate	moister
Thalictrum minus	warmer	drier
Thesium linophyllon	intermediate	drier
Thlaspi jankae	warmer	drier
Thymus pulegioides subsp. *pannonicus*	warmer	drier
Tragopogon dubius	intermediate	intermediate
Tragopogon orientalis	intermediate	intermediate
Trifolium alpestre	intermediate	drier
Trifolium montanum	intermediate	drier
Trifolium pratense	intermediate	moister

Trifolium repens	intermediate	intermediate
Urtica dioica	intermediate	moister
Valeriana officinalis subsp. *collina*	intermediate	drier
Verbascum austriacum	warmer	drier
Veronica austriaca	warmer	drier
Veronica chamaedrys	intermediate	intermediate
Vicia dumetorum	intermediate	intermediate
Vicia tetrasperma	intermediate	intermediate
Viola arvensis	intermediate	intermediate
Viola canina	intermediate	intermediate
Viola hirta	intermediate	drier
Waldsteinia geoides	warmer	intermediate

Table S3 Vector fitting results for the correlation of microclimate variables (T_{24}–RH_n) onto the non-metric multidimensional scaling (NMDS) ordinations. Significant correlations are indicated by bold *p* values

	Ants		Plants	
**Microclimate variables**	r^2	p	r^2	p
Mean daily temperature (T_{24})	0.45	**0.009**	0.65	**0.002**
Mean daytime temperature (T_d)	0.41	**0.018**	0.69	**0.001**
Mean night temperature (T_n)	0.32	0.052	0.20	0.174
Mean daily relative humidity (RH_{24})	0.27	0.086	0.56	**0.001**
Mean daytime relative humidity (RH_d)	0.31	**0.050**	0.57	**0.002**
Mean night relative humidity (RH_n)	0.03	0.818	0.10	0.429

Table S4 Comparisons of the occurrences of ant and plant functional groups for temperature requirements (warmer, intermediate and cooler) in different microhabitats (south-facing slopes, bottoms, north-facing slopes of dolines, and the plateau) in Bükk (Hungary) using the fitted mixed-effect models. The *p* values were corrected with the FDR (false discovery rate) method. Significant differences are indicated by bold *p* values

	**Warmer**		**Intermediate**		**Cooler**	
**Ants**						
Full model	*Chi*^{2}	*p*	*Chi*^{2}	*p*	*Chi*^{2}	*p*
	14.32	**0.003**	13.83	**0.003**	15.74	**0.001**
Pairwise comparisons	*z*	*p*	*z*	*p*	*z*	*p*
S-facing slopes vs. Bottoms	-3.17	**0.005**	1.37	0.256	2.4	**0.033**
S-facing slopes vs. N-facing slopes	-3.67	**0.001**	0.60	0.550	3.07	**0.006**
S-facing slopes vs. Plateau	-2.88	**0.008**	2.66	**0.031**	1.38	0.200
N-facing slopes vs. Bottoms	0.97	0.334	0.90	0.443	-1.13	0.260
N-facing slopes vs. Plateau	2.05	0.061	2.57	**0.031**	-3.15	**0.006**
Bottoms vs. Plateau	1.07	0.334	1.88	0.120	-2.03	0.064
**Plants**						
Full model	*Chi*^{2}	*p*	*Chi*^{2}	*p*	*Chi*^{2}	*p*
	50.75	< **0.001**	56.00	< **0.001**	47.08	< **0.001**
Pairwise comparisons	*z*	*p*	*z*	*p*	*z*	*p*
S-facing slopes vs. Bottoms	-5.21	< **0.001**	-3.15	**0.002**	-	-
S-facing slopes vs. N-facing slope	-5.22	< **0.001**	-0.05	0.961	-	-
S-facing slopes vs. Plateau	-1.22	0.268	3.49	**0.001**	-	-
N-facing slopes vs. Bottoms	0.22	0.828	-3.10	**0.002**	-0.49	0.626
N-facing slopes vs. Plateau	5.17	< **0.001**	3.54	**0.001**	-6.69	< **0.001**
Bottoms vs. Plateau	5.04	< **0.001**	6.83	< **0.001**	-6.31	< **0.001**

Table S5 Comparisons of the occurrences of ant and plant functional groups for moisture requirements (drier, intermediate and moister) in different microhabitats (south-facing slopes, bottoms, north-facing slopes of dolines, and the plateau) in Bükk (Hungary) using the fitted mixed-effect models. The *p* values were corrected with the FDR (false discovery rate) method. Significant differences are indicated by bold *p* values

	**Drier**		**Intermediate**		**Moister**	
**Ants**						
Full model	*Chi*^{2}	*p*	*Chi*^{2}	*p*	-	-
	14.32	**0.003**	16.10	**0.001**	-	-
Pairwise comparisons	*z*	*p*	*z*	*p*	-	-
S-facing slopes vs. Bottoms	-3.17	**0.005**	3.25	**0.002**	-	-
S-facing slopes vs. N-facing slopes	-3.67	**0.001**	3.47	**0.002**	-	-
S-facing slopes vs. Plateau	-2.88	**0.008**	3.70	**0.001**	-	-
N-facing slopes vs. Bottoms	0.97	0.334	-0.50	0.750	-	-
N-facing slopes vs. Plateau	2.05	0.061	-0.74	0.689	-	-
Bottoms vs. Plateau	1.07	0.334	-0.18	0.856	-	-
**Plants**						
Full model	*Chi*^{2}	*p*	*Chi*^{2}	*p*	*Chi*^{2}	*p*
	127.16	< **0.001**	13.17	**0.004**	67.65	< **0.001**
Pairwise comparisons	*z*	*p*	*z*	*p*	*z*	*p*
S-facing slopes vs. Bottoms	-7.72	< **0.001**	-1.75	0.162	6.30	< **0.001**
S-facing slopes vs. N-facing slope	-7.85	< **0.001**	0.27	0.786	7.00	< **0.001**
S-facing slopes vs. Plateau	-0.15	0.883	1.55	0.182	4.28	< **0.001**
N-facing slopes vs. Bottoms	-3.08	**0.003**	-2.01	0.132	-1.00	0.318
N-facing slopes vs. Plateau	8.22	< **0.001**	1.23	0.263	-5.29	< **0.001**
Bottoms vs. Plateau	7.80	< **0.001**	3.50	**0.003**	-3.96	< **0.001**

Table S6 Temperature and moisture requirements of ant species according to Czechowski et al. (2012), and the applied system

Temperature	
**Czechowski et al. (2012)**	**Applied system**
thermophiles	ants adapted to warmer conditions
mesothermo-thermophiles	
mesothermophiles	ants adapted to intermediate conditions
oligo-mesothermophiles	ants adapted to cooler conditions
oligothermophiles	
**Moisture**	
**Czechowski et al. (2012)**	**Applied system**
xerophiles	ants adapted to drier conditions
mesohygro-xerophiles	
mesohygrophiles	ants adapted to intermediate conditions
hygro-mesohygrophiles	absent in our study area
hygrophiles	

Table S7 Temperature and moisture requirements of plant species according to Borhidi (1995), and the applied system

**Temperature**	
**Borhidi (1995)**	**Applied system**
1 – species of subnival or supraboreal belt	absent in our study area
2 – species of alpine, boreal or tundra belt	
3 – species of subalpine or subboreal belt	
4 – species of montane needle-leaved forest or taiga belt	plants adapted to cooler conditions
5 – species of mesophilous broad-leaved forest belt	pants adapted to intermediate conditions
6 – species of submontane broad-leaved forest belt	
7 – species of thermophilous forest or woodland belt	plants adapted to warmer conditions
8 – species of sub-Mediterranean woodland and grassland belt	
9 – species of Eumediterranean evergreen belt	absent in our study area
**Moisture**	
**Borhidi (1995)**	**Applied system**
1 – plants of extremely dry habitats or bare rocks	absent in our study area
2 – xero-indicators on habitats with long dry period	plants adapted to drier conditions
3 – xero-tolerants, but eventually occurring on fresh soils	
4 – plants of semi-dry habitats	pants adapted to intermediate conditions
5 – plants of semi-humid habitats	
6 – plants of fresh soils	plants adapted to moister conditions
7 – plants of moist soils not drying out	
8 – plants of moist soils tolerating short floods	
9 – plants of wet, not well aerated soils	
10 – plants of frequently flooded soils	absent in our study area
11 – water plants with floating or partly emergent leaves	
12 – water plants, most wholly submersed in water	
